# Evaluation of risk factors for impulse control disorder in Parkinson’s disease in northern China

**DOI:** 10.3389/fnagi.2023.1257618

**Published:** 2023-11-21

**Authors:** Wenhua Ren, Yumeng Qi, Yan Liu, YaYun Yan, Xiaoqi Zheng, ShuXian Jin, Ying Chang

**Affiliations:** ^1^The Department of Neurology, China-Japan Union Hospital, Jilin University, Changchun, China; ^2^Departments of Biostatistics, Columbia University, New York, NY, United States; ^3^The Department of Neurology, Binzhou People Hospital, Shandong, China

**Keywords:** Parkinson’s disease, impulse control disorder, risk factors, hypersexuality, pathological gambling, compulsive shopping, binge eating

## Abstract

**Introduction:**

Impulse control disorder (ICD) is a common non-motor symptom of Parkinson’s disease (PD), but its risk factors are still controversial. This study aimed to determine the prevalence of ICD in northern China and analyze the risk factors associated with ICD, multiple ICDs, and four subtypes.

**Methods:**

A total of 285 PD patients were enrolled in this study. Each patient was screened using the Questionnaire for Impulse and Compulsive Control Disorders (QUIP). Stepwise regression analysis was performed to identify independent risk factors, and a prediction model was developed.

**Results:**

The prevalence of ICD in the study population was 11.6%. Stepwise regression analysis showed that ICD was associated with disease duration, motor symptoms, dyskinesia, depression, REM sleep behavior disorder (RBD) and cognitive decline; multiple ICDs were related to coffee history, motor symptoms, dyskinesia, depression, apathy and RBD. The prediction model demonstrated good performance with AUC values of 0.93, 0.88, and 0.66 on the balanced train set, balanced test set, and the original imbalanced data set, respectively.

**Conclusion:**

The risk factors for PD-ICD are complex and influenced by regional economic and cultural backgrounds. Clarifying these factors and developing predictive models can help to delay or even prevent the development of ICD through early screening and intervention.

## 1 Introduction

Parkinson’s disease (PD) is the second most common neurodegenerative disease after Alzheimer’s disease. The main clinical features are motor symptoms including bradykinesia, resting tremor, muscle rigidity, etc., and non-motor symptoms (NMS) such as olfactory disturbances, sleep disturbances, neuropsychiatric symptoms, constipation, pain, etc. NMS has no less negative impact on the patient’s quality of life than motor symptoms and also causes great distress to the patient’s caregivers, impulse control disorders (ICDs) being one of them. ICDs, as a psychiatric disorder motivated by a strong desire for self-gratification, are common comorbidities among PD patients treated with dopamine replacement therapy (DRT). The main ICDs are binge eating, compulsive shopping, pathological gambling, and hypersexuality. Because patients are ashamed to talk about their disease and even resist seeking treatment, ICDs often become unmanageable, which in turn severely impairs the patient’s quality of life, increases the burden of care on relatives, and often leads to divorce, unemployment, bankruptcy, and illegal behavior. Studying risk factors for ICDs is an important means for the early detection and prevention. Currently, research findings on risk factors are inconsistent, particularly regarding ICDs and apathy (Leroi et al., 2012; Tanaka et al., 2013), dyskinesia (Voon et al., 2011, 2017; Zhang et al., 2017), rapid eye movement sleep behavior disorder (RBD) (Scullin et al., 2013; Fantini et al., 2015; Baig et al., 2019; Lu et al., 2020), and dopamine receptor agonists (Tanaka et al., 2013; Phu et al., 2014; Zhang et al., 2017).

The prevalence of PD-ICD varies greatly across different regions, ranging from as low as 3.5% to as high as 49% (Fan et al., 2009; Garcia-Ruiz et al., 2014). There are also significant regional disparities in the prevalence of each subtype, with pathological gambling and compulsive shopping being more common among PD patients in the United States (Weintraub et al., 2010), but less common in India (Sarathchandran et al., 2013). Such geographical differences may be related to the socioeconomic and cultural backgrounds (Chiang et al., 2012; Vela et al., 2016). In this context, studying the risk factors of PD-ICD in different geographical areas may help to study its mechanism. At present, there is a lack of research on this topic in northern China, where the economy is slightly behind that of the south. Thus, in this study, we investigated the independent risk factors for ICD, multiple ICDs, and each subtype of ICDs (pathological gambling, binge eating, hypersexuality, and compulsive buying) in PD patients in northern China, respectively. And an attempt was made to establish a prediction model for PD-ICD. The aim was to provide a basis for the diagnosis and treatment of PD-ICD in northern China and to provide further support for subsequent related studies.

## 2 Materials and methods

### 2.1 Study design and sample

In this retrospective study, a total of 285 patients with PD treated at the Department of Neurology, China-Japan Union Hospital of Jilin University between September 2019 and December 2022 were enrolled. Inclusion criteria were: (1) All patients must meet the PD diagnostic criteria established by the Movement Disorder Society (MDS), as previously described (Postuma et al., 2015); (2) Patients were able to work with the researcher to complete the scale assessment. Exclusion criteria were: (1) Patients with secondary Parkinsonism; (2) Patients with Parkinson-plus syndrome. To ensure an authentic reflection of patients’ conditions, we administered the Questionnaire for Impulse and Compulsive Control Disorders (QUIP) to both patients and caregivers. In this study, we only analyzed patients whose responses in both questionnaires were entirely congruous. Furthermore, before the study began, we followed up the enrolled patients by telephone. QUIP is a tool that effectively screens for PD-ICD and is divided up into six sections: pathological gambling, binge eating, hypersexuality, compulsive buying, dopamine dysregulation syndrome, and punding. This study focused on ICDs, so only the first four sections were used, each containing two questions, and a positive screen for any one question was considered an ICD positive and was classified in the ICD group. If all questions in the four sections were negative, the patient was classified in the no ICD group (nICD group). In case of two or more positive aspects the patient was classified into the multiple ICD group.

### 2.2 Data collection and assessment

The demographic data collected included age, age of onset, gender, disease duration, education level, history of coffee, history of smoking, and history of alcohol consumption (Regular use of the above three for more than 1 year is considered meaningful). Clinical data collection: motor symptoms assessment included: the third part of the Unified Parkinson’s Disease Rating Scale of the Movement Disorder Society (MDS-UPDRS-III) and the Hoehn and Yahr grading of stages (H-Y grading) and the presence or absence of dyskinesia. Non-motor symptom assessment included: Hamilton Anxiety Scale (HAMA) (scores ≥ 14are positive) (Matza et al., 2010), Hamilton Depression Scale (HAMD) (scores ≥ 21are positive) (Hamilton, 1959), Apathy evaluation scale (AES) (scores≥14are positive) (Marin et al., 1991), RBD survey questionnaire (RBDSQ) (≥5 positive) (Stiasny-Kolster et al., 2007), Mini-mental State Examination (MMSE) and Montreal Cognitive Assessment (MoCA). We also counted the anti-Parkinsonian drugs taken by the patients and calculated the equivalent daily dose of levodopa. Meanwhile, the use of dopamine receptor agonists (DA), Pramipexole, Selegiline, and Amantadine was counted separately.

### 2.3 Statistical analysis

All statistical analyses were performed using Python 3.9.12.

To illustrate the distribution and characteristics of individual variables. The study incorporated univariate. For non-normal continuous variables, Mann–Whitney test was applied to compare the distribution of certain variable in ICD and nICD group; one ICD and multiple ICDs. For categorical data, Pearson’s chi-squared test was applied. Statistically significant differences were detected, with a significance level set at *p* < 0.05.

To reveal the relationship between variables and the presence of ICDs, a comprehensive analysis was conducted using stepwise regression, which was performed iteratively across six distinct models based on the presence of ICD, multiple ICD occurrences, and each specific ICD subtype as dependent variable. Stepwise regression, a widely adopted method for constructing regression models, involves a sequential selection process of predictor variables (Efroymson, 1960). Conventionally, the final model selection often hinges on the computation of *p*-values. However, relying solely on this approach can be overly simplistic and potentially overlook crucial nuances. In this study, a more robust and balanced approach was adopted through the use of the Akaike Information Criterion (AIC). The AIC is instrumental in mitigating the risks associated with both overfitting and underfitting, ensuring a more nuanced and fair model selection process.

To make the best predictive accuracy for the occurrence of ICDs, our study implemented the Synthetic Minority Oversampling Technique (SMOTE) to address imbalanced data. Subsequently, all variables were inclusively incorporated into the logistic regression model during the training phase. It is worth noting that our primary objective was to optimize predictive performance, and thus, the decision was made to retain all variables to capture the maximum information. To facilitate model evaluation and generalization to unseen data, we divided the dataset into two portions: 30% was randomly sampled as a test set, while the remainder was dedicated to the training set. During the training phase, a grid search combined with K-fold cross-validation played a pivotal role in fine-tuning the model’s hyperparameters. This approach not only enhanced model performance but also provided valuable insights into how the model would generalize to new and unseen data. Following model training and evaluation, the Area Under the Curve (AUC), a comprehensive metric quantifying the entire two-dimensional area beneath the Receiver Operating Characteristics (ROC) curve, was computed. This calculation was performed on the training set, test set, and the original dataset as a whole. The AUC serves as a robust indicator of the model’s predictive prowess across different datasets, offering a comprehensive assessment of its effectiveness.

## 3 Results

### 3.1 Univariate analysis

A total of 285 patients diagnosed with PD were included in the study, revealing a noteworthy prevalence of ICDs at 11.6%. Further exploration of ICDs revealed that the prevalence of multiple ICDs, including pathological gambling, binge eating, hypersexuality, and compulsive buying, stood at 4.2, 2.8, 7.0, 2.8, and 5.6%, respectively. In the comparison of the ICD group with the nICD group, significant differences were observed in the Disease duration, UPDRS III, Dyskinesia, HAMA, RBDSQ, LEDD, with *p*-values of 0.001, 0.035, 0.033, 0.017, 0.002, and 0.015, respectively (Table 1). No statistical differences were found between the Single-ICD and Multiple-ICD groups (Table 2).

**Table 1:** Univariate analysis: comparison of ICD and nICD groups.

	**ICD group *(n = 33)***	**nICD group *(n = 252)***	p−value
**Demographics characteristics**			
Gender (Male)	18 (54.55%)	112 (44.44%)	0.273
History of coffee (Yes)	2 (6.06%)	4 (1.59%)	0.092
History of smoking (Yes)	5 (15.15%)	38 (15.08%)	0.991
History of alcohol (Yes)	6 (18.18%)	35 (13.89%)	0.509
Age (years)	66.0 (59.0,69.5)	67.0 (59.0,71.0)	0.202
Education level (years)			0.394
0 ~ 6	5 (15.15%)	60 (23.81%)	
7 ~ 9	13 (39.39%)	77 (30.56%)	
10 ~ 12	6 (18.18%)	73 (28.67%)	
>12	9 (27.27%)	42 (16.67%)	
Disease duration (years)	8.0 (3.5,12.0)	5.0 (2.0,7.0)	0.001*
**Motor symptoms**			
UPDRS III	62.0 (40.0,79.0)	48.0 (34.0,65.8)	0.035*
H-Y grading			0.213
1	3	45	
1.5	2	28	
2	13	83	
2.5	1	11	
3	14	74	
4	0	5	
5	0	6	
Dyskinesia (Presence)	8 (24.24%)	28(11.11%)	0.033*
**Non-motor symptoms**			
HAMD (scores ≥21)	14 (42.42%)	87 (34.52%)	0.372
HAMA (scores ≥14)	21 (63.64%)	105 (41.67%)	0.017*
AES (scores ≥14)	26 (78.79%)	184 (73.02%)	0.479
RBDSQ (scores ≥5)	21 (63.64%)	91 (36.11%)	0.002*
MoCA	22.0 (17.0,24.0)	22.0 (17.3,25.0)	0.743
MMSE	27.0 (25.0,28.5)	27.0 (23.0,28.0)	0.517
**Medication**			
LEDD	500.0 (337.5,793.8)	375.0 (300.0,650.0)	0.015*
Dopamine receptor agonists (Yes)	22 (66.67%)	129 (51.19%)	0.094
Pramipexole (Yes)	16 (48.48%)	91 (36.11%)	0.167
Amantadine (Yes)	9 (27.27%)	44 (17.46%)	0.173
Selegiline (Yes)	2 (6.06%)	7 (2.78%)	0.311

Probability presented for qualitative variables. Median and interquartile range for quantitative variables. *p < 0.05.

**Table 2:** Univariate analysis: comparison of Single-ICD and Multiple-ICD groups.

	**Single-ICD group *(n = 21)***	**Multiple-ICD group *(n = 12)***	p−value
**Demographics characteristics**			
Gender (Male)	11 (52.38%)	7 (58.33%)	0.741
History of coffee (Yes)	0 (0.00%)	2 (16.67%)	0.054
History of smoking (Yes)	4 (19.05%)	1 (8.33%)	0.409
History of alcohol (Yes)	4 (19.05%)	2 (16.67%)	0.865
Age (years)	66.0 (59.0,68.5)	65.5 (55.0,72.3)	0.94
Education level (years)			0.791
0 ~ 6	3 (14.29%)	2 (18.18%)	
7 ~ 9	8 (38.10%)	5 (45.45%)	
10 ~ 12	4 (19.05%)	2 (18.18%)	
>12	6 (18.57%)	3 (17.17%)	
Disease duration (years)	9.0 (3.0,12.0)	6.0 (4.3,11.5)	0.523
**Motor symptoms**			
UPDRS III	64.0 (42.5,73.0)	52.5 (38.0,87.0)	0.837
H-Y grading			0.545
1	1 (4.76%)	2 (18.18%)	
1.5	1 (4.76%)	1 (9.09%)	
2	9 (42.56%)	4 (36.36%)	
2.5	1 (4.76%)	0 (0%)	
3	9 (42.56%)	5 (45.45%)	
4	0 (0%)	0 (0%)	
5	0 (0%)	0 (0%)	
Dyskinesia (Presence)	6 (28.57%)	2 (16.67%)	0.443
**Non-motor symptoms**			
HAMD (scores ≥21)	11 (52.38%)	3 (25.00%)	0.126
HAMA (scores ≥14)	15 (71.43%)	6 (50.00%)	0.218
AES (scores ≥14)	15 (71.43%)	11 (91.67%)	0.171
RBDSQ (scores ≥5)	15 (71.43%)	6 (50.00%)	0.218
MoCA	22.0 (17.5,24.0)	22.0 (15.5,24.0)	0.895
MMSE	27.0 (24.0,28.5)	27.5 (26.3,28.8)	0.677
**Medication**			
LEDD	500.0 (412.5,793.8)	400.0 (300.0,875.0)	0.293
Dopamine receptor agonists (Yes)	15 (71.43%)	7 (58.33%)	0.443
Pramipexole (Yes)	10 (47.62%)	6 (50.00%)	0.895
Amantadine (Yes)	8 (38.10%)	1 (8.33%)	0.065
Selegiline (Yes)	1 (4.76%)	1 (8.33%)	0.679

Probability presented for qualitative variables. Median and interquartile range for quantitative variables. *p < 0.05.

### 3.2 Stepwise regression

To avoid multicollinearity, we calculated pairwise Spearman rank correlation coefficients. In this study, age and age of onset, as well as H-Y grading and UPDRS III scores, were highly correlated (coefficient higher than 0.8). Therefore, we removed age and UPDRS-III scores and performed stepwise logistics regression with the remaining variables, and Table 3 demonstrates the summary of the model. Details are shown in Supplementary Tables S1–S6.

**Table 3:** Correlation between different risk factors and PD-ICD, multiple ICD, and subtypes of ICD.

	Gender	Age of onset	Disease duration	History of coffee	HAMA	HAMD	AS	RBDSQ	MMSE	MOCA	Selegiline	Amantadine
ICD			+		+			+	+			
Multiple ICD		−		+			+					−
Binge Eating	+	−	+				+		+			
Compulsive Shopping		−	+		+	−		+				−
Hypersexuality	+	−		+							−	
Pathological gambling			+	+						−		−

The “+” indicates a positive correlation between the factor and the risk of disease, while the “–” indicates a negative correlation between the factor and the risk of disease.

The dependent variables appearing in the table are the independent risk factors for the predictor variables. And the sign and magnitude of the coefficients (β) indicate the relationship between the predictor and dependent variables. For example, in the case of ICD when other variables remain constant, if the dependent variable is a continuous variable and increases by one unit, or if the dependent variable is a categorical variable and changes classification, the likelihood of the patient having an ICD increases by a factor of e^β^.

### 3.3 Logistic regression for prediction

The utilization of stepwise regression procedures in data mining has been a topic of contention due to potential biases in tests and the risk of creating oversimplified models (Rencher and Pun, 1980; Roecker, 1991). Additionally, it’s worth noting that the incidence of ICD in the dataset was relatively low, hovering around 4%, which indicated a significant data imbalance. This imbalance could potentially result in the development of highly accurate yet less informative models.

**Figure 1:**
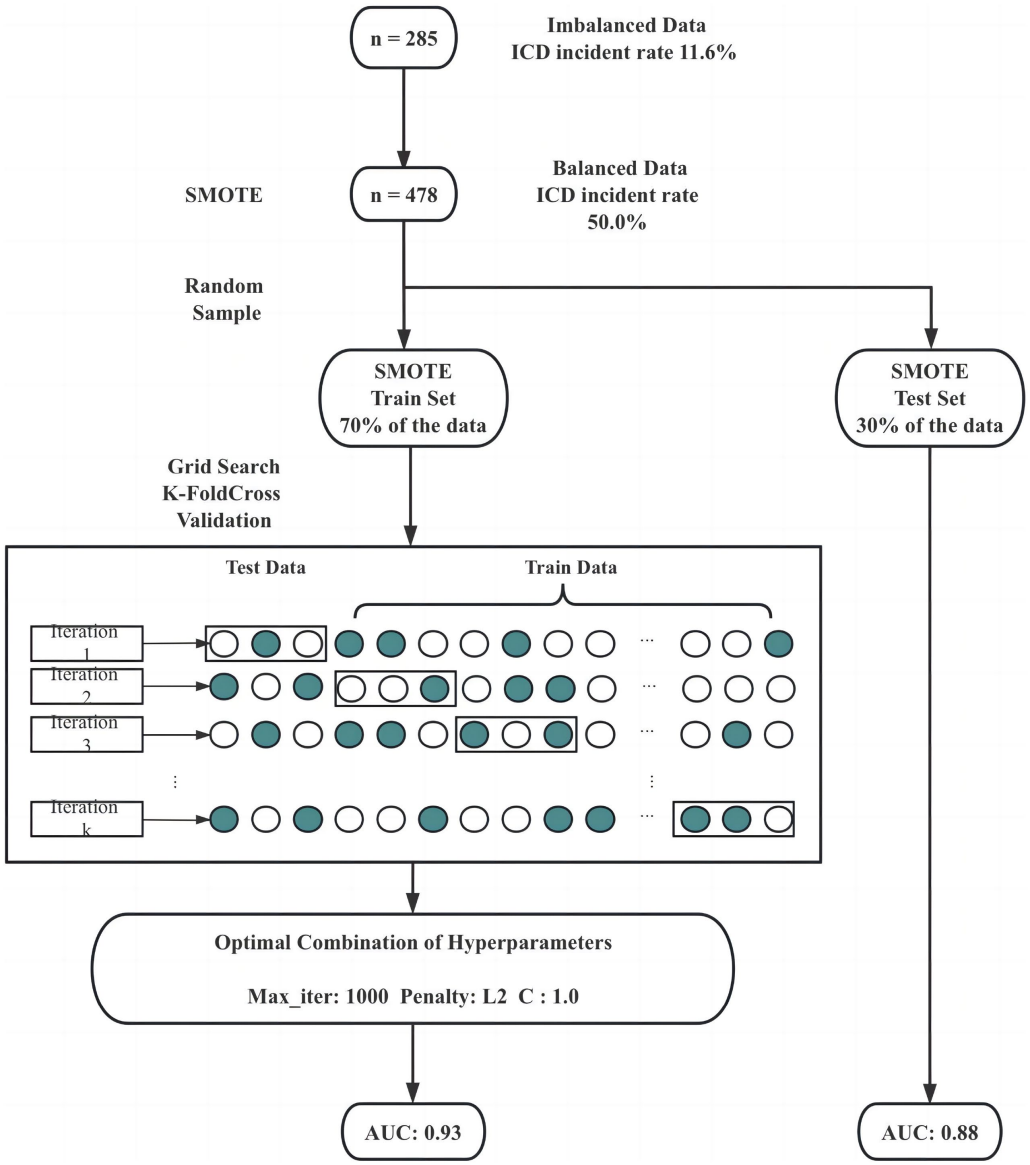
Flowchart for building the predictive model.

**Figure 2:**
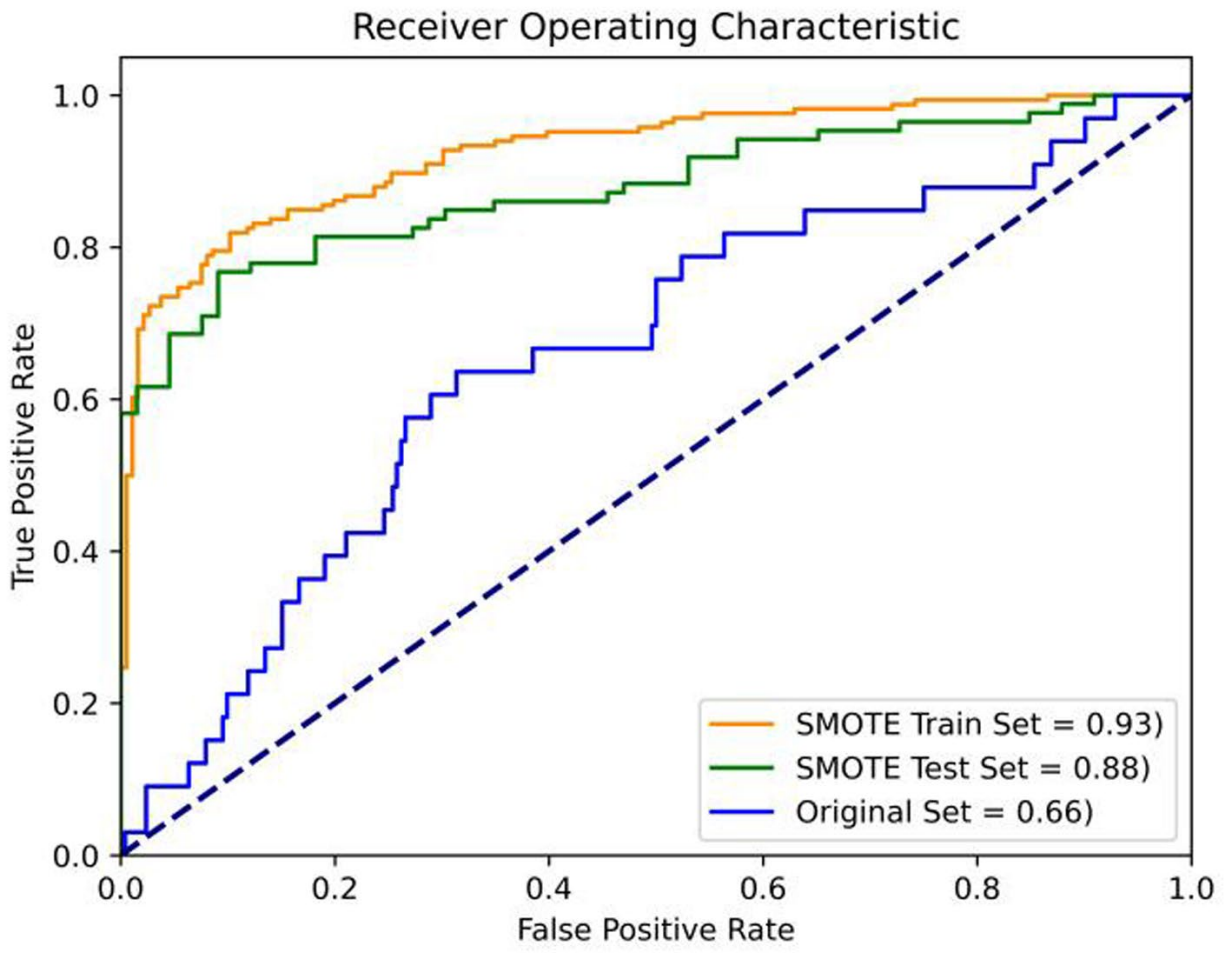
Verification of prediction model. Orange: the balanced train set (AUC = 0.93); Green: balanced test set (AUC = 0.88); Blue: the original imbalanced data set (AUC = 0.66).

To address these challenges, our study took a strategic approach. First, we balanced the dataset using the Synthetic Minority Oversampling Technique (SMOTE). SMOTE works by selecting examples that are close in the feature space, drawing a line between the examples in the feature space and drawing a new sample at a point along that line. The study applied SMOTE to generate synthetic examples until the ratio of ICD and nICD is 1:1. Subsequently, the data was randomly divided into two sets, with the larger share (70%) allocated for model training. The process of model selection involved grid search and K-fold cross-validation to identify the optimal combination of hyperparameters, including maximum iteration time, the inverse of regularization strength, and penalty norm. In our study, we employed a value of k equal to 5, and this entire process is depicted in Figure 1. Following grid search, the hyperparameters of the logistic model were determined, with a maximum iteration time (max_iter) set to 1,000, an inverse regularization strength (C) of 1, and an L2 penalty. To evaluate the binary classifier’s performance in distinguishing between classes, we calculated the Area Under the Curve (AUC) on the balanced training set, the balanced test set, the unbalanced original dataset. Remarkably, the AUC values stood at 0.93, 0.88 and 0.66, respectively, underscoring the model’s exceptional predictive capability and generalization prowess (as shown in Figure 2).

## 4 Discussion

In this retrospective study, we examined the incidence of ICD in the northern region of China (which is economically slightly less developed compared to the southern region) and analyzed the risk factors associated with ICD. This study showed that the prevalence of PD-ICD in northern China was 11.6 and 4.2% for multiple ICDs, with the highest incidence of binge eating (7.0%), followed by compulsive buying (5.6%), and pathological gambling (2.8%) and hypersexuality (2.8%) being rare. Stepwise regression analysis showed that independent risk factors for ICD included disease duration, anxiety, RBD and cognitive decline. Risk factors for multiple ICDs consisted of age of onset, the coffee history, apathy, and the use of amantadine. Risk factors for binge eating comprised gender, age of onset, disease duration, apathy and cognitive decline. Risk factors for compulsive shopping included age of onset, disease duration, anxiety, depression, RBD and the use of amantadine. Risk factors for hypersexuality included age of onset, gender, the coffee history and the use of selegiline. Risk factors for pathological gambling comprised disease duration, the coffee history, cognitive decline and the use of amantadine. A prediction model with good predictive and generalization capabilities was also developed in this study.

The incidence rate of ICD varies significantly across regions. One possible reason for this disparity is the utilization of different screening methods, such as the QUIP questionnaire with a higher positivity rate. Even under the same conditions of using the QUIP questionnaire, there are distinct variations in incidence rates among countries. For example, in this study, the prevalence in northern China is 11.6%, while it is 28% in Japan (Tanaka et al., 2013), 15.4% in Malaysia (Garcia-Ruiz et al., 2014), 34.8% in Finland (Joutsa et al., 2012), and 18.4% in Brazil (Valença et al., 2013). These discrepancies may be attributed to genetic and other factors. Genetic differences among populations may contribute to variations in ICD incidence rates. Sociocultural factors, economic conditions, and educational levels, may also impact incidence rates. For instance, the prevalence of pathological gambling ranges from 3.0 to 8.8% in Europe (Joutsa et al., 2012; Perez-Lloret et al., 2012; Callesen et al., 2014; Antonini et al., 2017) and 5% in North American countries (Sarathchandran et al., 2013). In this study, the prevalence in northern China was only 2.8%, which may be related to the fact that gambling is legal in some countries (e.g., Finland) and China has a strict anti-gambling policy. Additionally, the treatment approach for PD may influence ICD incidence rates, as different regions employ diverse medication protocols.

It’s worth noting that even within the same country, there are disparities in the incidence rates of ICD among different regions. In economically more developed southern regions, the incidence rate of ICD appears to be lower, such as 4.15% in Shanghai (Wang et al., 2016), 7% in Hong Kong (Auyeung et al., 2011), and 9.15% in Zhejiang (Xu et al., 2021). This might be attributed to fewer specialized doctors for PD in northern China, a delay in early diagnosis and treatment, and a lower rate of early medical consultation among patients. It could also be associated with the implementation of varied screening methods.

Consistent with previous studies, we found that demographic factors such as male gender, earlier age of onset, and longer disease duration were associated with PD-ICD (Faouzi et al., 2021; Cao et al., 2022). A history of coffee consumption emerged as a risk factor for multiple ICDs, pathological gambling, and hypersexuality. This suggests that individuals with PD-ICD may be prone to coffee addiction or that long-term exposure to addictive substances like caffeine may increase susceptibility to PD-ICD.

There is controversy regarding the association between PD-ICD and motor symptoms. Some reports have suggested a link between ICD and motor symptoms in PD patients. A prospective study found an association between ICD and lower H-Y grading (Bastiaens et al., 2013). Previous research has also found that PD patients with ICD exhibit more complex motor symptoms such as fluctuations, on/off phenomena, dystonia and freezing (Leroi et al., 2012; Callesen et al., 2014). However, other studies have indicated that UPDRS-III scores and H-Y staging are not related to the occurrence of ICD, and our study also showed such results (Weintraub et al., 2013; Marinus et al., 2018; Cao et al., 2022). The reasons for the inconsistent study results are unclear and require further research.

The relevance of ICD to dyskinesia is still controversial. Both dyskinesia and ICD are behavioral side effects in terms of motor and NMS caused by long-term dopaminergic drug therapy. Both occur mainly involving the dopamine system and the basal ganglia circuit, and both are associated with the early onset of PD and dopamine-like drug application. However, more differences than similarities are found between the two. For example, dyskinesia mainly involves the motor circuits of the dorsal striatum and motor cortex, and dyskinesia is often associated with the severity of PD and the use of levodopa (Jiménez-Urbieta et al., 2015). Females have a greater risk for dyskinesia than males (Zappia et al., 2005; Van Gerpen et al., 2006). ICDs, however, mainly involve the ventral striatum and limbic circuits of the prefrontal cortex and are associated with higher doses of DA. ICDs are more common in males (Zappia et al., 2005; Van Gerpen et al., 2006). Two teams found PD who experienced levodopa-induced dyskinesias tended to exhibit ICD more frequently and with greater severity compared to patients without LID (Ramírez Gómez et al., 2017; Simoni et al., 2020). However, dyskinesia was negatively associated with the development of ICD, which indicated that among PD patients, those without ICD symptoms had a significantly higher prevalence of dyskinesia compared to PD patients with ICD (Biundo et al., 2017). In our study, we did not find a correlation between dyskinesia and ICD, which is consistent with the findings in Cao L’s research (Cao et al., 2022). The correlation between the two needs to be supported by further studies.

Researchers have demonstrated interest in the association between PD-ICD and NMS, particularly regarding anxiety, depression, and cognitive functioning. Marín-Lahoz et al. (2019) highlighted depression as a risk factor for ICD, while Pineau et al. (2016) and Erga et al. (2017) found associations between both depression and anxiety with ICD, aligning with our own results. However, Mack et al. (2013) and Piray et al. (2014) found no such connections between depression, anxiety, and ICD. Tessitore et al. (2016) discovered that the ICD group performed worse executive function in comparison to the nICD group. Nevertheless, some studies have yielded different results, with two showing comparable performance between the two groups (Piray et al., 2014; Pineau et al., 2016), and one study even observing ICD group performed better (Siri et al., 2010). Our study demonstrated a significant correlation between cognitive function and ICD. Further research is necessary to confirm the associations between ICD and anxiety, depression, and cognitive function.

The current understanding regarding the relationship between RBD and PD-ICD remains uncertain. A multicentre study found that baseline RBD was an independent predictor of ICD development over time (Cao et al., 2020). A meta-analysis involving 2,781 patients with PD showed that PD patients with comorbid RBD had a 2-fold increase in the incidence of ICD (Lu et al., 2020). In contrast, Fahd Baig, Mark J. Kelly et al. noted that RBD was not associated with an increased risk of ICD (Baig et al., 2019). The present study shows that RBD is an independent risk factor for ICD. There is no clear understanding of the mechanism by which RBD is associated with ICD. RBD is considered a precursor to synucleinopathies, and ICD is a common comorbidity for those treated with dopaminergic replacement therapy (DRT). RBD usually occurs earlier than ICD. It has been suggested that the mesocorticolimbic dopaminergic pathway may be a key point in the connection between ICD and RBD (Lu et al., 2020). This pathway includes the ventral striatum (namely the ventral tegmental area and the nucleusaccumbens), the amygdala, and the hippocampus, which plays an important role in reward, reinforcement learning, and impulse control. The long-term dopaminergic treatment causes alterations in pre- and postsynaptic dopamine levels, which may lead to ventral striatum sensitization and, in turn, to the development of ICD. The brainstem region responsible for REM sleep regulation is also a component of the limbic system (Wilson et al., 2009), which may also play a role in the development of RBD (Iranzo et al., 2006). Additionally, related research indicates that the correlation between the two may be associated with functional connectivity changes between the limbic striatum and posterior cortical regions (Marques et al., 2021).

Historically, apathy and ICD have been considered as contrasting phenomena. Previous studies have suggested that ICD is a reward-seeking behavior that is the result of dopamine overdose, whereas apathy is mainly manifested as reduced interest in activities and is a reward-deficient behavior that is associated with low-dose dopamine medication (Leroi et al., 2012). However, as research progressed, it was found that ICD also occurred in patients not on dopamine medication and apathy occurred in patients on high doses of dopamine medication (Scott et al., 2020). Leroi et al. (2012) pointed out that apathy is a potential risk factor for ICD and may play a role in the development of hypersexuality. Related studies have discovered that ICD and apathy occur in the same brain area in patients with frontotemporal dementia (Lansdall et al., 2017; Passamonti et al., 2018). The mechanism by which apathy and ICD can coexist remains unclear, and there may be a common neuroregulatory network, and their occurrence is associated with disruption of the neuroregulatory network (Petitet et al., 2021). This study shows that apathy is a risk factor for multiple ICD and binge eating, providing evidence for subsequent studies of the association between the two.

In terms of medication, unlike most previous studies, the presence or absence of DA use was not an independent risk factor for ICD in this study. Possible reasons for this are (1) regional DA is mostly pramipexole, but it is expensive and patients take small doses; (2) regional physicians are conservative in their use of the DA. Different receptor agonists have different risks of ICD due to their different affinities for D2 and D3 receptors. DAs with high affinity for D3 receptors (Pramipexole, Ropinirole) are more likely to develop ICD, whereas DAs with low affinity for D3 receptors (Rotigotine) have a lower risk of developing ICD (Torti et al., 2019). ICD prevalence analysis between different DA drugs was not performed in this study. The reason for this is that pramipexole is reimbursed by medical insurance in northern China, is easy to purchase, and is used by many patients, while rotigotine and others are difficult to purchase and have few users.

Apart from DA, there is ongoing controversy surrounding the relationship between other anti-Parkinsonian medications and ICD. A study by Weintraub et al. demonstrated an increased incidence of ICD with amantadine (Weintraub et al., 2010), while a smaller study suggested that oral amantadine reduced the occurrence of ICD (Thomas et al., 2010). In our study, we observed a negative correlation between taking amantadine and the presence of multiple ICDs, compulsive shopping and pathological gambling. Garcia-Ruiz et al. identified an association between ICD and rasagiline (Catalán et al., 2013; Garcia-Ruiz et al., 2014), whereas our study found a negative association between taking rasagiline and hypersexuality.

When considering all medications collectively, our study did not find LEDD to be a risk factor for ICD. However, we did observe a higher LEDD in patients with ICD, leading us to hypothesize that the prevalence of ICD may increase when multiple anti-PD drugs are introduced at a certain dosage. Therefore, for individuals at high risk of ICD, it remains crucial to monitor the elevation in LEDD when using alternative medications in lieu of DA.

Meanwhile, we built a prediction model using the existing data and provided the model-building method. The AUC values show that the model has good prediction and generalization ability, and through continuous optimization training, this model is likely to improve our ability to diagnose PD-ICD in northern China.

## 5 Limitations

The limitations of this study are as follows. Firstly, being a single-center study with a small sample size might result in insufficient sample representativeness, thereby limiting the generalizability of the research findings. Secondly, despite our efforts to mitigate underreporting by utilizing both patient and caregiver responses on the QUIP questionnaire, as well as conducting follow-up assessments prior to the study, it is important to acknowledge that the topic of ICD is sensitive, potentially leading to patient reluctance or inability to provide accurate information. Lastly, while the logistic regression model allows for exploring the relationship between risk factors and ICD, it cannot establish causality. Additionally, it can only investigate the known and speculated risk factors, potentially overlooking unknown or unconsidered underlying factors. Therefore, future research will involve multicentre studies and longitudinal investigations to ascertain causal relationships.

## 6 Conclusion

The risk factors of PD-ICD are numerous and intricately intertwined. These factors are closely associated with the regional economic and cultural background. Our study has found that in the diagnosis and treatment of PD-ICD in northern China, special attention should be given to the following factors: male, earlier age of onset, longer disease duration, history of coffee consumption, anxiety, apathy, RBD, and poor cognitive function. A thorough understanding of patients’ basic information is crucial in elucidating the relationship between these factors and ICD. Establishing a region-specific predictive model for ICD is highly beneficial for the early screening and intervention in high-risk PD patients. With such a model, we can predict which patients are more likely to develop ICD, allowing us to delay or even prevent its occurrence.

## Data availability statement

The raw data supporting the conclusions of this article will be made available by the authors, without undue reservation.

## Ethics statement

This study has been approved by the Ethics Committee of China-Japan Union Hospital. Due to the study being retrospective and the data being anonymous, the requirement for a informed consent statement was waived.

## Author contributions

WR: Data curation, Formal analysis, Resources, Writing – original draft. YQ: Formal analysis, Methodology, Software, Visualization, Writing – review & editing. YL: Data curation, Investigation, Writing – original draft. YY: Validation, Writing – review & editing. XZ: Data curation, Validation, Writing – review & editing. SJ: Validation, Writing – review & editing. YC: Conceptualization, Project administration, Supervision, Writing – review & editing.
