# Supplementary material for: Evaluation of risk factors for impulse control disorder in Parkinson’s disease in northern China
Source: Front Aging Neurosci. 2023 Nov 21;15:1257618. doi: 10.3389/fnagi.2023.1257618 (PMC10702947; doi:10.3389/fnagi.2023.1257618)
Supplement: Supplementary file 1 [file Table_1.DOCX]

Table 1. Logistic regression of influencing factors of ICD with PD.

|  | Coefficients | SD | Odds Ratio | 95% Confidence interval |
| --- | --- | --- | --- | --- |
| (Intercept) | -0.2450 | 0.1198 | 0.7827 | (0.6189, 0.9899) |
| Disease Duration | 0.0166 | 0.0047 | 1.0168 | (1.0074, 1.0262) |
| MMSE | 0.0079 | 0.0042 | 1.0079 | (0.9997, 1.0162) |
| HAMA | 0.0670 | 0.0395 | 1.0692 | (0.9896, 1.1553) |
| RBDSQ | 0.0957 | 0.0389 | 1.1005 | (1.0196, 1.1877) |
| Notes: Variables are selected by stepwise logistic regression which minimized Akaike information criterion (AIC = 144.6). Confidence interval is calculated for odds ratio. | | | | |

Table 2. Logistic regression of influencing factors of multiple ICD with PD.

|  | Coefficients | SD | Odds Ratio | 95% Confidence interval |
| --- | --- | --- | --- | --- |
| (Intercept) | 0.1260 | 0.0783 | 1.1343 | (0.973, 1.3225) |
| Age of Onset | -0.0019 | 0.0012 | 0.9981 | (0.9957, 1.0006) |
| Coffee | 0.2982 | 0.0810 | 1.3474 | (1.1496, 1.5794) |
| Amantadine | -0.0474 | 0.0306 | 0.9537 | (0.8982, 1.0127) |
| AES | 0.0407 | 0.0265 | 1.0415 | (0.9888, 1.097) |
| Notes: Variables are selected by stepwise logistic regression which minimized Akaike information criterion (AIC = -113.2). Confidence interval is calculated for odds ratio. | | | | |

Table 3. Logistic regression of influencing factors of binge eating with PD.

|  | Coefficients | SD | Odds Ratio | 95% Confidence interval |
| --- | --- | --- | --- | --- |
| (Intercept) | 0.0088 | 0.1607 | 1.0089 | (0.7362, 1.3825) |
| Age of Onset | -0.0029 | 0.0017 | 0.9971 | (0.9938, 1.0005) |
| Disease Duration | 0.0068 | 0.0042 | 1.0068 | (0.9986, 1.015) |
| Gender | 0.0572 | 0.0303 | 1.0589 | (0.9979, 1.1236) |
| MMSE | 0.0052 | 0.0034 | 1.0052 | (0.9985, 1.0119) |
| AES | 0.0515 | 0.0345 | 1.0528 | (0.984, 1.1265) |
| Notes: Variables are selected by stepwise logistic regression which minimized Akaike information criterion (AIC = 28.4). Confidence interval is calculated for odds ratio. | | | | |

Table 4. Logistic regression of influencing factors of compulsive shopping with PD.

|  | Coefficients | SD | Odds Ratio | 95% Confidence interval |
| --- | --- | --- | --- | --- |
| (Intercept) | 0.1850 | 0.1012 | 1.2032 | (0.9868, 1.4671) |
| Age of Onset | -0.0031 | 0.0015 | 0.9969 | (0.994, 0.9999) |
| Disease Duration | 0.0059 | 0.0038 | 1.0059 | (0.9985, 1.0133) |
| Amantadine | -0.0533 | 0.0365 | 0.9481 | (0.8826, 1.0185) |
| HAMD | -0.0671 | 0.0346 | 0.9351 | (0.8738, 1.0006) |
| HAMA | 0.0726 | 0.0332 | 1.0753 | (1.0076, 1.1476) |
| RBDSQ | 0.0642 | 0.0286 | 1.0663 | (1.0081, 1.1278) |
| Notes: Variables are selected by stepwise logistic regression which minimized Akaike information criterion (AIC = -33.0). Confidence interval is calculated for odds ratio. | | | | |

Table 5. Logistic regression of influencing factors of hypersexuality with PD.

|  | Coefficients | SD | Odds Ratio | 95% Confidence interval |
| --- | --- | --- | --- | --- |
| (Intercept) | 0.1081 | 0.0605 | 1.1142 | (0.9896, 1.2544) |
| Age of Onset | -0.0016 | 0.0010 | 0.9984 | (0.9964, 1.0003) |
| Gender | 0.0303 | 0.0189 | 1.0308 | (0.9934, 1.0696) |
| Coffee | 0.3378 | 0.0675 | 1.4019 | (1.2281, 1.6003) |
| Selegiline | -0.1123 | 0.0557 | 0.8938 | (0.8013, 0.9969) |
| Notes: Variables are selected by stepwise logistic regression which minimized Akaike information criterion (AIC = -235.7). Confidence interval is calculated for odds ratio. | | | | |

Table 6. Logistic regression of influencing factors of pathological gambling with PD.

|  | Coefficients | SD | Odds Ratio | 95% Confidence interval |
| --- | --- | --- | --- | --- |
| (Intercept) | 0.1041 | 0.0425 | 1.1097 | (1.0209, 1.2062) |
| Coffee | 0.3215 | 0.0659 | 1.3792 | (1.2122, 1.5693) |
| Disease Duration | 0.0036 | 0.0025 | 1.0036 | (0.9987, 1.0086) |
| Amantadine | -0.0361 | 0.0250 | 0.9645 | (0.9183, 1.013) |
| MoCA | -0.0047 | 0.0017 | 0.9954 | (0.992, 0.9988) |
| Notes: Variables are selected by stepwise logistic regression which minimized Akaike information criterion (AIC = -239.3). Confidence interval is calculated for odds ratio. | | | | |
